# Molecular epidemiology and multidrug resistance of *Mycobacterium tuberculosis* complex from pulmonary tuberculosis patients in the Eastern region of Ghana

**DOI:** 10.1016/j.heliyon.2021.e08152

**Published:** 2021-10-09

**Authors:** Benjamin D. Thumamo Pokam, Dorothy Yeboah-Manu, Daniel Amiteye, Prince Asare, Prisca Wabo Guemdjom, Nchawa Yangkam Yhiler, Samuel Nii Azumah Morton, Stephen Ofori-Yirenkyi, Roger Laryea, Roger Tagoe, Anne Ebri Asuquo

**Affiliations:** a Department of Medical Laboratory Science, Faculty of Health Sciences, University of Buea, Buea, Cameroon; b Bacteriology Department, Noguchi Memorial Institute for Medical Research, University of Ghana, Legon, Accra, Ghana; c Department of Biomedical Engineering, All Nations University College, Koforidua, Ghana; d Department of Public Health, Faculty of Health Sciences, University of Buea, Buea, Cameroon; e Department of Allied Health, Biaka University Institute, Buea, Cameroon; f Eastern Regional Hospital, Koforidua, Ghana; g Department of Medical Laboratory Science, Faculty of Allied Medical Sciences, College of Medicine, University of Calabar, Calabar, Nigeria

**Keywords:** Tuberculosis, MDR, Cameroon sub-lineage, Eastern region, Ghana

## Abstract

**Background:**

Tuberculosis **(**TB) and drug-resistant TB (DR-TB) continue to persist as a serious public health challenges in Ghana. Although several research has evaluated the drug resistance of *Mycobacterium tuberculosis* complex (MTBc) strains across the country, there is a paucity of data on its magnitude as well as the various lineages circulating in the Eastern region of Ghana.

**Objective:**

This study therefore evaluated the distribution of the various lineages of MTBc in the Eastern region of the country and the associated drug resistance.

**Materials and methods:**

One hundred and forty-three (143) patients with pulmonary TB attending the Eastern Regional Hospital, Koforidua/Ghana were included in the study. The BACTEC MGIT 960 tube media was used for both sputum culture and drug susceptibility of streptomycin (STR), isoniazid (INH), rifampicin (RIF) and Ethambutol (ETH). Isolates were initially typed using IS*6110*, followed by large sequence polymorphisms analysis and spoligotyping.

**Results:**

The majority [108 (75.5%)] of the 143 patients were male gender and the 45–54 years [46 (32.2%)] age range had the highest frequency. Forty-one (28.7%) of the 143 isolates were *IS6110* negative. Of the 102 spoligotyped isolates, the main sub-lineages included 45 (44.1%) Cameroon and 23 (22.5%) Ghana. SITs 61 and 53 represented the major cluster with 22/102 (21.6%) and 13/102 (12.7%) isolates respectively, while 59/65 (90.8%) isolates belonged to Lineage 4 with 27/65 (41.5%) LAM10_CAM. MDR-TB occurred in 26/79 (32.9%) isolates, and was not associated with neither gender [20/58 (34.5%) male vs 6/21 (28.6%) female, OR = 1.31; 95%CI, 0.44–3.92; p = 0.624)] nor age. No association was found between MDR-TB and the major sub-lineages [8/25 (32%) Cameroon (OR = 0.94; 95%CI, 0.34–2.59; p = 0.920) and 5/11 (45.5%) Ghana (OR = 1.87; 95%CI, 0.51–6.80; p = 0.489)], or previously treated [8/23 (34.8%), OR = 0.89; 95%CI, 0.32–2.48; p = 0.823)] patients.

**Conclusion:**

Despite the serious threat posed by MDR in the study area, no sub-lineage was shown to be associated with drug resistance. Nonetheless, a sustained surveillance of drug resistance pattern is advocated. A lower proportion of *M. africanum* was observed in the Eastern region of Ghana and will require further evaluation.

## 1 Introduction

Tuberculosis (TB) is considered a worldwide threat and it ranks behind Human Immunodeficiency Virus (HIV) as the second cause of death from an infectious disease [1]. The causative agent of TB is a group of closely related acid-fast bacteria known as the *Mycobacterium tuberculosis* complex (MTBc). The agent of TB is transmissible via aerosolisation of droplet nuclei containing particles of *Mycobacterium tuberculosis* (MTB) released from the lungs of patients with pulmonary disease [2, 3]. The past century has witnessed a significant drop in the rate of TB infection in industrialised regions. Nevertheless, the disease still persists as a major public health problem particularly in resource poor countries of the sub-Saharan region in spite of the availability of effective antituberculosis chemotherapy for over several decades [3]. The highest incidence of TB cases in 2019 occurred in the South-East Asian and the African regions with 44% and 25% of new cases respectively [1]. An incidence of about five hundred thousand cases of rifampicin-resistant TB (RR-TB) occurred in 2018, out of which about 80% were multidrug resistant TB (MDR -TB). Moreover, 3.4% of new TB cases and 18% of formerly treated cases had MDR-TB or RR-TB globally [4].

The TB control efforts have been handicapped for quite a while by the poor understanding of the diverse strains of mycobacteria circulating across the world [5], eluded by classical epidemiologists for decades. In recent years, molecular methodologies have allowed the analyses of the genetic material of MTBc, including the analysis among others of the insertion element IS*6110*, the polymorphism within the direct repeat (DR) locus and using mycobacterial interspersed repetitive units (MIRUs). This has led tremendously to the molecular typing of the mycobacteria, and brought insights into the different lineages of MTBc. This in turn has contributed to the detection of new cases, relapse, and reinfection by new strains as well as improving the control of the disease in different countries [6]. The molecular epidemiology of tuberculosis can therefore detect the rates of active transmission, differentiate between recent or previous infection as well as recurrent or exogenous reinfection [3].

Although the direct detection of MTBc from clinical specimens can be achieved by molecular biological methods within a shorter time [7, 8], culture still represents the definitive method for the diagnosis of tuberculosis. The rapid and reliable techniques for culture detection of acid-fast bacilli (AFB) were developed [9, 10], enabling mycobacterial culture not only with the slow conventional solid media, but available broth-based methods. The use of radiometric semi-automated BACTEC 460TB system, considered as a the “gold standard” [11], which permitted significantly earlier detection of mycobacteria, has since been replaced by non-radiometric technologies for growth and detection of AFB such as the fluorimetric Mycobacteria Growth Indicator Tube (MGIT) [12]. Equally, the noninvasive and non-radiometric BACTEC MGIT-960 system is currently available and operates with the same technology as the manual MGIT. It is a fully automated system that utilizes the fluorescence of an oxygen sensor for the detection of the growth of mycobacteria in culture and the shortest times to detection obtained is 13.3 days [13]. An inbuilt quality control system ensures a precise and reliable operation, providing results as positive/negative as well as numerical growth units.

TB still constitutes a public health challenge in Ghana. In 2018, The country recorded and notified to WHO, 13,978 (32% of expected cases) incident cases of TB [4]. The report of the national TB prevalence survey in 2013, estimated a prevalence of 264/100,000 population for all forms of TB and bacteriological prevalence of 356/100,000 population with smear-positive rate of 105/100,000 population [14]. Several studies have reported on the drug resistance patterns of the isolates and lineages distribution in the country. However, data are missing on the impact of MTB drug resistance and strains distribution in the Eastern region in Ghana. This study therefore evaluated the pulmonary TB in patients from one of the 10th region and environs of Ghana using liquid culture technique as well as drug resistance patterns and genetic diversity of isolates recovered from the Eastern region of the country.

## 2 Materials and methods

### 2.1 Study setting and ethical considerations

This was a cross sectional study carried out between January and December 2017, and including 143 diagnosed pulmonary TB patients seeking healthcare at the Eastern Regional Hospital (formerly Koforidua General Hospital) located in the New Juaben Municipality of Koforidua, the capital of the Eastern region of Ghana. The regional hospital serves as a TB diagnostic referral center for the 21 districts of the region, as well as some sharing boundaries’ regions as the Ashanti and Greater Accra. The approval for the study was initially granted by the Scientific and Technical Committee and later by the ethical committee of the Institutional Review Board (IRB) of Noguchi Memorial Institute for Medical Research (NMIMR) [FWA 00001824; IRB 00001276; NMIMR-IRB CPN 007/16–17; IORG 0000908].

### 2.2 Sputum culture using BD BACTEC™ MGIT™ 960

The collected sputum samples underwent the standard digestion and decontamination by means of N-acetyl-l-cysteine–2% NaOH procedure (BBL™ MycoPrep***™***; Becton Dickinson). Briefly, the pellet was re-suspended after discarding the supernatant in sterile phosphate buffer to a final volume of 2ml. A smear preparation was carried out using one part of the mixture and stained using the standard Zhiel Neelsen techniques, while another part was inoculated in one BACTEC MGIT 960 tube (0.5 ml) media supplemented with the antibiotic mixture polymyxin B, amphotericin B, nalidixic acid, trimethoprim, and azlocillin (PANTA) and growth supplement (Becton Dickinson). The BACTEC MGIT 960 tubes were incubated at 37 °C for up to 42 days in the BACTEC MGIT 960 apparatus which monitor for increased fluorescence (corresponding to the amount of oxygen consumed by the organisms in the inoculated specimens) every 60 min and positive signal development automatically (https://www.finddx.org › mgit_manual_nov2006).

### 2.3 Genotyping of the isolates

The emulsified media was subjected to IS*6110* and RD deletion typing identifying RD 1, 4, 9, 12, 702, 711 using the methods and primers described elsewhere [16]. Further testing using spoligotyping following manufacturer's instructions (Isogen Bioscience, The Netherlands) was carried as described by Kamerbeek *et al.* [17], on a membrane using the 43-spacer, and the MTBc lineages were grouped as previously defined [18]. Positive controls included *M. tuberculosis* H37Rv and *M. bovis* BCG DNAs, while SDW served as negative control.

### 2.4 BACTEC MGIT 960 drug sensitivity tests

Susceptibility testing to antimycobacterial drugs was carried out as described earlier using BACTEC MGIT 960 [15]. The lyophilised vial containing the low concentration of each drug (Becton-Dickinson) was diluted using 4 ml of sterile distilled water (SDW). The following final low drug concentrations were obtained by pipetting 0.1 ml of this dilution into an MGIT tube to obtain 1.0 μg/ml streptomycin (STR), 0.1 μg/ml isoniazid (INH), 1.0 μg/ml rifampicin (RIF), and 5.0 μg/ml ethambutol (ETH). Control tubes contained broth without drugs and 1:100 inoculum dilutions were set up as microbial controls to ensure growth in the controls tubes. Higher concentrations were equally prepared for STR, INH, and ETH by dissolving each high-concentration of lyophilized drugs in 2 ml of SDW. Following the transfer of 0.1 ml into the MGIT tube, the final high drug concentrations of 4.0 μg/ml for STR, 0.4 μg/ml for INH, and 7.5 μg/ml for ETH were obtained.

### 2.5 Data analysis

The SPSS version 20 (IBM, Chicago, IL, USA) analyzed the association between the variables and p-values less than 0.05 were considered statistically significant. The comparison of proportions was carried out using the Pearson's Chi-square while the Fisher's exact-test was used when more than 20% of the expected frequencies were less than 5. The spoligotype patterns in a binary format were analyzed (Supplementary material I) using the SpolDB4 database/MIRU-VNTR*plus* [19].

## 3 Results

### 3.1 Patient characteristics and distribution of the genetic families

The study included 143 participants [108 (75.5%) male and 35 (24.5%) female] aged between 17 and 89 years (mean = 46.09 years). The majority 46 (32.2%) of the 143 patients were aged between 45 to 54 years, followed by 28 (19.6%) within 35–44 years and 9 (6.3%) who were less than 24 years. One hundred and eight (75.5%) patients were newly diagnosed while 35 (24.5%) had undergone treatment previously.

**Figure 1:**
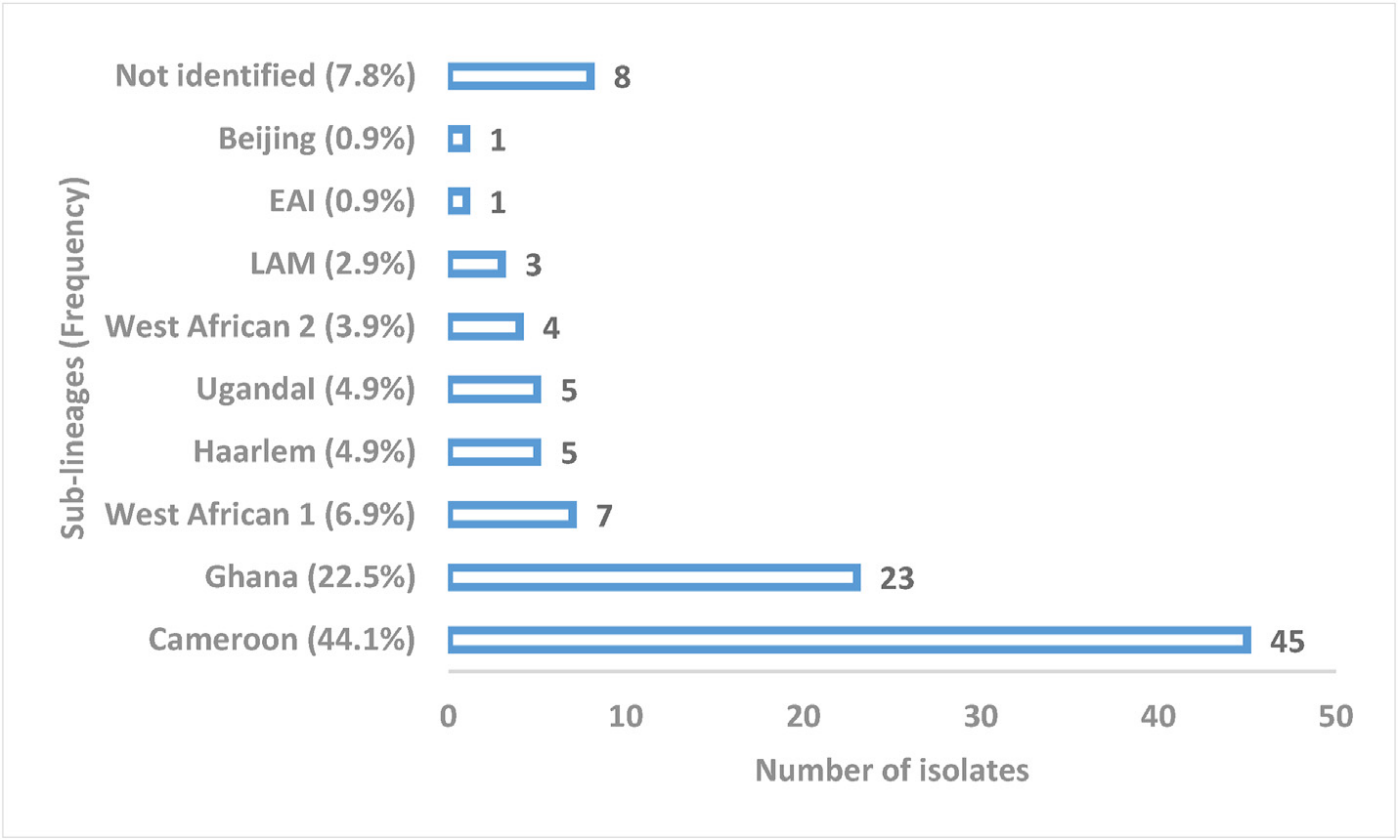
Sub-lineages distribution of the isolates in the Eastern Region of Ghana.

Of the 143 isolates from the liquid BACTEC MGIT 960, 41 (28.7%) were *IS6110* negative. One hundred and two isolates were spoligotyped, including 83 (81.4%) classified as *M. tuberculosis* sensu stricto, 11 (10.8%) as *M. africanum,* and 8 (7.8%) not identified. The 102 isolates were subdivided into 9 sub-lineages and included 45 (44.1%) Cameroon, followed by 23 (22.5%) Ghana, 7 (6.9%) West African 1, 5 (4.9%) Haarlem, 5 (4.9%) UgandaI, 4 (3.9%) West African 2, 3 (2.9%) LAM, 1 (0.9%) EAI, 1 (0.9%) Beijing and 8 (7.8%) not identified (Figure 1).

Fifty-one different patterns were obtained from the 102 spoligotyped isolates. Eighteen Shared International Types (SITs) were identified and 37 orphans. SITs 61 and 53 were the most represented cluster with 22 (21.6%) and 13 (12.7%) isolates respectively. Among the 65 identified lineages, 59 (90.8%)] belonged to Lineage 4 [27 (41.5%) LAM10_CAM, 21 (32.3%) T1, 5 (7.7%) H3, 3 (4.6%) LAM9, 2 (3.1%) T2 and 1 (1.5%)T1 genotypes], 1 (1.5%) to lineage 2 (the Beijing), 1 (1.5%) to lineage 3 (EAI5) and 4 (6.2%) to lineage 5 [1 (1.5%) AFRI and 3 (4.6%) AFRI_2] (Table 1).

**Table 1:** Distribution of genotypes, sublineages, spoligotypes international types (SITs) and spoligotypes patterns of 102 *Mycobacterium tuberculosis* complex isolates in the Eastern Region of Ghana.

Lineages	Genotypes	Sublineages	SITs	Spoligotypes patterns	No. of isolates	Frequency (%)
L2 (n = 1)	BEIJING	Beijing	1		1	1
L3 (n = 1)	EAI5	EAI	342		1	1
L4 (n = 59)	H3	Haarlem	1243		1	1
	H3	Haarlem	50		4	3.9
	LAM9	LAM	42		3	2.9
	LAM10_CAM	Cameroon	61		22	21.6
	LAM10_CAM	Cameroon	772		3	2.9
	LAM10_CAM	-	57		1	1
	LAM10_CAM	Cameroon	838		1	1
	T1	Ghana	53		13	12.7
	T1	-	205		1	1
	T1	Ghana	393		1	1
	T1	Ghana	1166		5	4.9
	T1	-	1626		1	1
	T2	UgandaI	848		2	1.9
	T3	-	504		1	1
L5 (n = 4)	AFRI	West African 1	330		1	1
	AFRI_2	West African 1	331		3	2.9
	Not classified				37	36.3
	Total				102	

### 3.2 Drug susceptibility of the isolates

Drug susceptibility test was carried out on 79 isolates, of which 26 (32.9%) were pansusceptible (susceptible to all 4 drugs), while resistance to at least one drug irrespective of the combinations occurred in 45(57%), 29 (36.7%), 14 (17.7%) and 40 (50.6%) INH, RIF, ETH and STR respectively. MDR (concomitant resistance to INH and RIF) occurred in 26/79 (32.9%) [ (3.8%) INH and RIF, 12 (15.2%) INH, RIF and STR, 11 (13.9%) to all the four drugs INH, RIF, ETH and STR] polyresistant isolates (Table 2).

**Table 2:** Drug susceptibility patterns of the isolates in the study area.

Drugs	MTBc n = 79 (%)
**Pansusceptible**	26 (32.9)
**Any resistance**	
INH	45 (57)
RIF	29 (36.7)
ETH	14 (17.7)
STR	40 (50.6)
**Monoresistance**	
INH	6 (7.6)
RIF	1 (1.3)
ETH	1 (1.3)
STR	4 (5.1)
**Polyresistance**	
INH + RIF	3 (3.8)
INH + ETH	2 (2.5)
INH + STR	11 (13.9)
RIF + STR	2 (2.5)
INH + RIF + STR	12 (15.2)
INH + RIF + ETH + STR	11 (13.9)
**MDR – TB** (Any polyresistance INH + RIF)	26 (32.9)

MTBc = *Mycobacterium tuberculosis* complex, INH = Isoniazid, RIF = Rifampicin, ETH = Ethambutol STR = Streptomycin.

### 3.3 Association of multi-drug resistance with gender, age, sub-lineages and previous treatment

The association between gender, age, sub-lineages, previously treated patients and MDR - TB is shown in Table 3. MDR – TB was neither associated with gender [20/58 (34.5%) male vs 6/21 (28.6%) female (OR = 1.31; 95%CI, 0.44–3.92; p = 0.624)] nor age of the studied subjects. However, though not significant, MDR-TB was mainly found in patients aged below 24 years and those above 64 years [4/9 (44.4%), OR = 1.75; 95%CI, 0.43–7.14) vs 4/6 (40%), OR = 1.42; 95%CI, 0.36–5.56; p = 0.722].

**Table 3:** MDR association between gender, age, sub-lineages and previously treated patients.

	Number of isolates tested n = 79	Non-MDR-TB (%) n = 53	MDR-TB (%) n = 26	Odds Ratio (95% CI)	P-value
**Gender**				1.31 (0.44–3.92)	0.624
Male	58	38 (65.5)	20 (34.5)		
Female	21	15 (71.4)	6 (28.6)		
**Age (Years)**					
<25	9	5 (55.6)	4 (44.4)	1.75 (0.43–7.14)	0.467
25–34	13	8 (61.5)	5 (38.5)	1.34 (0.39–4.59)	0.749
35–44	13	10 (76.9)	3 (23.1)	0.56 (0.14–2.24)	0.528
45–54	21	14 (66.7)	7 (33.3)	1.03 (0.36–2.96)	0.582
55–64	13	10 (76.9)	3 (23.1)	0.56 (0.14–2.24)	0.528
>64	10	6 (60)	4 (40)	1.42 (0.36–5.56)	0.722
**Sub-lineages**					
Beijing	1	0	1 (100)	-	0.329
Cameroon	25	17 (68)	8 (32)	0.94 (0.34–2.59)	0.920
EAI	1	1 (100)	0	-	0.671
Ghana	11	6 (54.5)	5 (45.5)	1.87 (0.51–6.80)	0.489
Haarlem	1	0	1 (100)	-	0.329
LAM	1	1 (100)	0	-	0.671
UgandaI	3	3 (100)	0	-	0.547
West African 1	6	5 (83.3)	1 (16.7)	0.38 (0.04–3.47)	0.658
West African 2	4	2 (50)	2 (50)	2.13 (0.28–16.0)	0.595
Undefined	26	18 (69.2)	8 (30.8)	0.86 (0.32–2.37)	0.777
**Treatment status**				0.89 (0.32–2.48)	0.823
Newly diagnosed	56	38 (67.9)	18 (32.1)		
Previously treated	23	15 (65.2)	8 (34.8)		

Of the 25 Cameroon sub-lineages, 8 (32%) were MDR (OR = 0.94; 95%CI, 0.34–2.59; p = 0.920), while 5/11 (45.5%) Ghana sub-lineage were equally MDR (OR = 1.87; 95%CI, 0.51–6.80; p = 0.489). The others MDR included 2/4 (50%) West African 2, 1/6 (16.7%) West African 1, 1/1 (100%) Haarlem and 1/1 (100%) Beijing. There was no statistical association between the sub-lineages and MDR in the study area.

The comparison of drug resistance between newly diagnosed and previously treated patients showed that 8 (34.8%) isolates among the 23 previously treated patients were MDR - TB (OR = 0.89; 95%CI, 0.32–2.48; p = 0.823), while 18/56 (32.1%) were from newly diagnosed patients.

## 4 Discussion

Rapid diagnosis and drug sensitivity of mycobacterial infections is important to shorten the time to detection and management of the disease to avoid its dissemination especially in communities. The BACTEC-MGIT 960 system is an automated and non-radiometric culture system using a continuous monitoring of the consumption of O_2_ [13] that meets that requirement.

Drug resistance of MTBc is a significant threat especially among previously treated patients. This study recorded an MDR of 32.9% which is lower than the 36% recorded earlier in the country [20] and similar to the 32% recorded in Nigeria [21]. However, this is higher than reported in a more recent study carried out in the Ghana capital's Accra, which recorded 27.7% MDR-TB among previously treated patients [22]. About a quarter (24.5%) of participants included in our study were previously treated (relapse, treatment failure, default) patients.

Considering drug resistance individually, this study recorded a high resistance to INH (57%) and STR (51%). Several studies in Ghana have pointed out the rising prevalence of STR resistance especially amongst TB cases that have been previously treated [20, 23], as well as its wide occurrence in the country [24]. Several explanations of the high resistance to STR have alluded to its long use not only for TB treatment, but also for several different pathologies, thus leading to its abuse especially in African countries where over the counter purchase of drugs without control and regulations are common practice. The increase in primary STR resistance transmission is also assumed to emerge from reactivation of latent TB infection [22].

High prevalence of both INH (12.5%) and STR (20.6%) has been reported in a nationwide survey in Ghana probably because both drugs have been used in the first line anti-TB drug regimen for a longer period than RIF and ETH [25]. This is nevertheless lower than our study which also included previously treated patients. An elevated prevalence of primary resistance to INH has been detected (23%) in the country, probably as a result of high defaulting rate (37%) indicating poor TB control [26], and calling for a sustained reinforcement of the Directly observed treatment, short-course (DOTS) system in a bid to treat the disease better. The increasing MDR- TB resistance in new patients with strains resistant to one or two of the potent drugs prior to treatment cannot be over emphasized [25].

A wide study on drug-resistance in eight West African countries including Ghana has shown a high prevalence of MDR strains in either new (6 %) or retreatment patients (35 %), with the highest prevalence amongst retreatment patients in Bamako - Mali (59 %) and two Nigerian sites (Ibadan and Lagos respectively with 39 % and 66 %). MDR has been shown to be spreading actively amongst 32 % of new patients in Lagos and 35% in Ghana [27], suggesting in line with our study that drug-resistance prevalence in the country poses a serious public health threat.

The genotyping of the MTBc in the Eastern region of Ghana has shown that 81.4% isolates were *Mycobacterium tuberculosis* sensu stricto and is in line with previous studies carried out in Northern and Southern (79.1%) as well as the South-western (80.25%) part of country earlier [28, 29]. Surprisingly, this study reported a lower prevalence (10.8%) of *M. africanum* (6.9% of West African 1 and 3.9% of West African 2). Two previous studies have shown that *M. africanum* represented up to 19.75% and 17.1% of all human TB in the country [29, 30]. Although the absence of a decline in the occurrence of *M. africanum* was observed recently (20.2%) in the country [28], the probability that these strains are following the same decreased trend observed earlier in Cameroon might not be excluded [31]. It could thus be hypothesized that the Cameroon sub-lineage (44.1% in the study area) might be gradually and successfully replacing the Africanum lineages in the Eastern region of Ghana. Opinions are currently divided on the contribution of *M. africanum* to the burden of TB in the west African region and research on its real impact has been proposed earlier [16, 32]. Nevertheless, the reduced virulence of *M. africanum*, its slower rate to progression to active disease as well as its longer latency compared to MTB [33, 34] might be factors possibly leading to its decrease over time.

The Euro-American lineage 4 was the most predominant one encountered in the Eastern region of Ghana and included the Cameroon (44.1%) and Ghana (22.5%) genotype. A similar study to ours previously undertaken in the country showed that up to 36 % of the Cameroon sub-lineage was recorded in South-Western Ghana and about 17% by the Ghana sub-lineage [29]. A similar trend has been observed in a neighbouring West African country where up to 21% were of the Ghana genotype, buttressing the dominance of this lineage in the region and thus most likely to be involved in community transmission as shown in Mali with the Cameroon genotype [35]. Although we cannot infer active transmission of the Cameroon and Ghana genotype clusters in our study considering the low discriminatory power of spoligotyping, the presence of the Cameroon lineage has been largely shown to be present in West African countries including Ghana, Nigeria, Burkina Faso and Cameroon, and could be a reflection of intra-regional free movement of population within the Economic Community of West African States [36]. The other MTBc lineages belonging to especially to Lineage 4 have been largely observed in the country as well as within other African regions [16, 30, 32]. Lineage 4 has been shown to be geographically widespread [18], and high recent transmission rate of this lineage has been shown to occur recently in Ghana [37].

This study has shown that 32% and 45.5% of Cameroon and Ghana sub-lineages respectively were MDR. The MDR-TB linked to Cameroon genotype has been shown to be widespread in Nigeria [38]. Equally, drug resistance in Ghana has been linked to the Ghana genotype [28] and the latter has been found to be an important cause of MDR-TB in Bamako – Mali [35]. Mali and Nigeria being neighbouring countries of Ghana, the clonal expansion of MDR in the sub-region with likely risk of community transmission cannot be excluded. Phylogenetic analysis has shown previously the implication of a sub-clade of the Ghana genotype belonging to the T1 family to be more likely associated with drug resistance compared to other genotypes [35, 39]. The Beijing strains as well as its association with MDR reported in this study has earlier been described in the country [28]. The dominance of the Beijing strains has been recorded in South Africa, with up to 19.2% of all TB cases, while in West African region, Guinea accounted for 5.3% and Gambia for 5.2% [36]. This is not unconnected with the current influx migration of Asian population to the African region as well as increase trade relations of Africans returning from travel to China for business, leading to the spread of this Lineage 2 in the continent. High prevalence of MDR–TB [40] as well as increase transmission has been linked with L2–Beijing [41, 42] and its being increasingly reported in Africa [43, 44].

## 5 Conclusion

MDR-TB strains in the Eastern region of Ghana is particularly worrisome considering the possibility of its community transmission. The Cameroon and Ghana genotypes were the predominant clade encountered and a slight decrease of *M. africanum* was observed in the study area. Although there was no obvious association between the sub-lineages and drug resistance, the presence and dissemination of the Beijing strain in the study area might pose a serious threat in the future considering the development and increase drug resistance in the region. Newer strategies and policies should be elaborated for efficient control of the surge of MDR in the region. Active surveillance which may include prior screening and evaluation of risky individuals coming from areas in the west African sub-region as well as foreign countries across the world with known MDR-TB strains is advocated. This will help to prevent the consequence and threat on naive indigenous populations in the long term in a country where widespread diagnosis as well as laboratory infrastructure still requires some improvement to efficiently curb the propagation of MDR-TB.

## Declarations

### Author contribution statement

Benjamin D. Thumamo Pokam: Conceived and designed the experiments; Performed the experiments; Analyzed and interpreted the data; Contributed reagents, materials, analysis tools or data; Wrote the paper.

Dorothy Yeboah-Manu: Conceived and designed the experiments; Contributed reagents, materials, analysis tools or data; Wrote the paper.

Daniel Amiteye: Conceived and designed the experiments; Performed the experiments; Contributed reagents, materials, analysis tools or data.

Prince Asare: Performed the experiments; Contributed reagents, materials, analysis tools or data.

Prisca Wabo Guemdjom: Analyzed and interpreted the data; Wrote the paper.

Nchawa Yangkam Yhiler: Analyzed and interpreted the data; Contributed reagents, materials, analysis tools or data.

Samuel Nii Azumah Morton, Stephen Ofori-Yirenkyi, Roger Laryea, Roger Tagoe: Performed the experiments.

Anne Ebri Asuquo: Conceived and designed the experiments; Wrote the paper.

### Funding statement

This work was supported by the 10.13039/100000865Bill and Melinda Gates Foundation to BDTP under the Postdoctoral and Postgraduate Training in Infectious Diseases Research awarded to the 10.13039/501100012017Noguchi Memorial Institute for Medical Research (Global Health Grant number OPP52155).

### Data availability statement

Data included in article/supp. material/referenced in article.

### Competing interest statement

The authors declare no conflict of interest.

### Additional information

No additional information is available for this paper.
