# Supplementary figures and images for: Molecular epidemiology and multidrug resistance of Mycobacterium tuberculosis complex from pulmonary tuberculosis patients in the Eastern region of Ghana
Source: Heliyon. 2021 Oct 9;7(10):e08152. doi: 10.1016/j.heliyon.2021.e08152 (PMC8551511; doi:10.1016/j.heliyon.2021.e08152)

**Supplementary material I: Phylogenetic tree of 102 MTBc isolated in the Eastern region of Ghana**


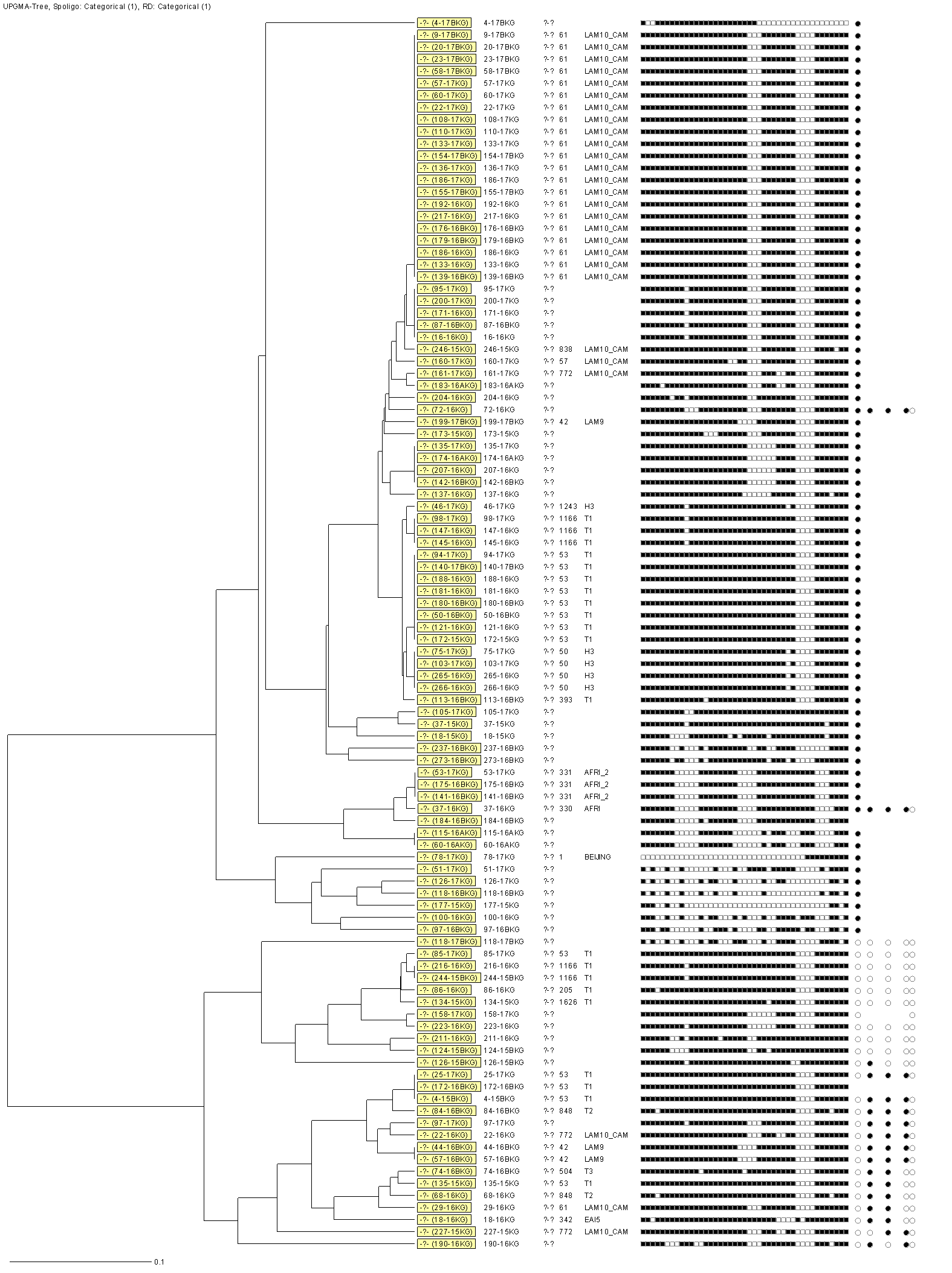

Supplement: Supplementary material I [file mmc1.docx]
